# Association between renal sympathetic denervation and arterial stiffness: the ASORAS study

**DOI:** 10.1097/HJH.0000000000003361

**Published:** 2023-01-19

**Authors:** Victor J.M. Zeijen, Lida Feyz, Isabella Kardys, Marcel L. Geleijnse, Nicolas M. Van Mieghem, Felix Zijlstra, Melvin Lafeber, Rob J. Van Der Geest, Alexander Hirsch, Joost Daemen

**Affiliations:** a Department of Cardiology; b Department of Internal and Vascular Medicine, Erasmus University Medical Center, Rotterdam; c Department of Radiology, Leiden University Medical Center, Leiden; d Department of Radiology and Nuclear Medicine, Erasmus University Medical Center, Rotterdam, The Netherlands

**Keywords:** blood pressure, kidney, MRI, pulse wave analysis, sympathectomy, ultrasonography, vascular stiffness

## Abstract

Renal sympathetic denervation (RDN) reduces blood pressure (BP). However, one out of three patients does not exhibit a significant BP response to the therapy. This study investigates the association between noninvasive vascular stiffness indices and RDN-mediated BP reduction.

**Methods:**

In this prospective, single-arm pilot study, patients with systolic office BP at least 140 mmHg, mean 24-h systolic ambulatory blood pressure (ABP) at least 130 mmHg and at least three prescribed antihypertensive drugs underwent radiofrequency RDN. The primary efficacy endpoint was temporal evolution of mean 24-h systolic ABP throughout 1-year post RDN (measured at baseline and 3–6–12 months). Effect modification was studied for baseline ultrasound carotid–femoral and magnetic resonance (MR) pulse wave velocity (PWV), MR aortic distensibility, cardiac MR left ventricular parameters and clinical variables. Statistical analyses were performed using linear mixed-effects models, and effect modification was assessed using interaction terms.

**Results:**

Thirty patients (mean age 62.5 ± 10.7 years, 50% women) with mean 24-h ABP 146.7/80.8 ± 13.7/12.0 mmHg were enrolled. Following RDN, mean 24-h systolic ABP changed with −8.4 (95% CI: −14.5 to −2.3) mmHg/year (*P* = 0.007). Independent effect modifiers were CF-PWV [+2.7 (0.3 to 5.1) mmHg/year change in outcome for every m/s increase in CF-PWV; *P* = 0.03], daytime diastolic ABP [−0.4 (−0.8 to 0.0) mmHg/year per mmHg; *P* = 0.03], age [+0.6 (0.2 to 1.0) mmHg/year per year of age; *P* = 0.006], female sex [−14.0 (−23.1 to −5.0) mmHg/year as compared with men; *P* = 0.003] and BMI [+1.2 (0.1 to 2.2) mmHg/year per kg/m^2^; *P* = 0.04].

**Conclusion:**

Higher CF-PWV at baseline was associated with a smaller reduction in systolic ABP following RDN. These findings could contribute to improve identification of RDN responders.

## INTRODUCTION

Whereas adequate antihypertensive therapy has demonstrated to reduce cardiovascular risk in hypertensive patients, blood pressure (BP) control is achieved in merely 40% of all patients receiving medical treatment [1–4].

To date, sympathetic renal denervation (RDN) is the most widely studied invasive antihypertensive treatment modality. Six randomized sham-controlled trials demonstrated that RDN significantly lowers systolic ambulatory blood pressure (ABP) by 4–8 mmHg in the absence of major adverse events [5–10]. Yet, about one in three patients does not experience a significant reduction (> 5 mmHg) in systolic ABP following RDN [6,7,10–12]. Previous studies have identified a variety of patient-related and procedure-related predictors as well as pharmacological predictors of response [13–20]. Nevertheless, the reproducibility of these findings is limited, warranting further research on the topic.

Arterial stiffness has emerged as an independent cardiovascular risk factor, which is highly prevalent among hypertensive patients [21,22]. Arterial stiffness hampers the physiological transition of pulsatile flow into steady flow in the microcirculation [23]. Both hypertension and arterial stiffness are strongly related to increased sympathetic activity [24–26]. Arterial wall stiffness can be assessed by measuring pulse wave velocity (PWV), local aortic distensibility (AoD), or by the ambulatory arterial stiffness index (AASI) [27,28].

In previous research, invasively measured PWV, magnetic resonance (MR) AoD and AASI were identified as independent predictors of the BP response to RDN [29–33]. Moreover, reductions in PWV, AoD, and cardiac remodeling were observed post RDN [34–37]. In contrast to the extensively studied role of invasive PWV, little data is available on noninvasively measured arterial stiffness indices in relation to RDN response [32]. Consequently, the aim of this study was to assess effect modification of the BP response following RDN by noninvasive arterial stiffness parameters at baseline, including PWV, in patients with resistant hypertension, as well as to assess how these parameters change over time following RDN.

## MATERIALS AND METHODS

### Study design

This study was a prospective, single-arm pilot study performed in the Erasmus University Medical Center (Rotterdam, The Netherlands).

### Study population

Adult patients were eligible when their systolic office blood pressure (OBP) was at least 140 mmHg and mean 24-h systolic ABP was at least 130 mmHg despite the use of three or more antihypertensive drugs (including at least one diuretic). A lower number of antihypertensive drugs was allowed only in case of documented intolerance to three or more classes of antihypertensive drugs. Exclusion criteria were pregnancy, renal artery anatomy ineligible for RDN, estimated glomerular filtration rate (eGFR) less than 45 ml/min per 1.73 m^2^, known secondary causes of hypertension (except obstructive sleep apnea syndrome) and any contra-indications for MR imaging. Anatomical exclusion criteria involved renal artery diameter less than 4 mm, renal artery length less than 20 mm, renal artery stenosis at least 50%, renal artery aneurysm or previous renal artery intervention. All patients provided written informed consent. The center's local ethics committee approved the study protocol and the study was conducted in accordance with the Declaration of Helsinki.

### Blood pressure measurement

Standardized OBP measurement was performed using the Omron M10-IT device (OMRON Healthcare Europe, Hoofddorp, The Netherlands) [38]. Measurements were performed in both arms, and two additional readings were obtained from the arm with the initial highest reading. OBP was calculated as the average of the last two measurements. Twenty-four hour ABP measurements were performed using the Spacelabs 90217A device (Spacelabs Healthcare, Snoqualmie, Washington, USA). AASI was calculated as 1 − the regression slope of diastolic versus systolic ABP measurements, reflecting a parallel relationship between arterial stiffness and AASI increase [28].

### MRI

Patients were scanned on a clinical 1.5T MR imaging system (Discovery MR450 or SIGNA Artist, both GE Healthcare, Milwaukee, Wisconsin, USA) using a dedicated cardiac/anterior array coil. The cardiovascular magnetic resonance (CMR) protocol for left-ventricular (LV) mass, volumes and function involved a retrospectively ECG-gated balanced steady-state free precession (SSFP) cine imaging with breath-holding. A contiguous stack of LV short-axis views was obtained from base to apex. Typical scan parameters were slice thickness 8.0 mm, slice gap 2.0 mm, repetition time/echo time (TR/TE) 3.7/1.6 ms, flip angle 75°, field of view (FOV) 380 × 340 mm, acquired matrix 160 × 192 and 24 phases per cardiac cycle. Functional analysis was performed on short-axis images by manually drawing epicardial and endocardial contours in end-systolic and end-diastolic phase. Papillary muscles were included in the mass and excluded from the volume. LV end-diastolic and end-systolic volume and LV mass were measured. Consequently, these parameters were used to calculate LV stroke volume, ejection fraction and cardiac output. Maximal LV wall thickness was also measured at the short-axis views. Volumes and mass were indexed by body surface area (BSA) as calculated using the formula from Du Bois and Du Bois [39].

Similarly, the protocol for MR-PWV measurement involved a free-breathing, retrospectively ECG-gated 2D phase-contrast flow sequence. The slice plane was positioned at the level of the pulmonary artery perpendicular to the ascending and descending aorta. Typical scan parameters were FOV 420 × 315 mm, matrix size 256 × 256, slice thickness 5.0 mm, flip angle 30°, TR/TE 4.5/2.4 ms, views per segment 1, NEX 2, velocity encoding value 200 cm/s, true temporal resolution ∼11 ms and number of reconstructed phases 100 per cardiac cycle. Aortic arch PWV was calculated by dividing the distance between ascending and descending aorta by the delta time of the systolic wave front [40]. The onset of the systolic wave front was calculated as the time point at the intersection between the tangent of the velocity upslope and the constant diastolic velocity. The length of the aortic segment between the ascending and descending aorta was quantified from a sagittal angulated multislice 3D retrospectively ECG-gated balanced SSFP acquisition during breath-hold. Typical scan parameters were slice thickness 8.0 mm, slice gap – 4.0 mm, TR/TE 2.8/1.3 ms, flip angle 45°, FOV 360 × 290 mm and acquired matrix 192 × 160.

Subsequently, MR-AoD was measured using a retrospectively ECG-gated 2D balanced SSFP sequence during breath-hold. This scan was obtained perpendicular to the ascending aorta at the level of the pulmonary trunk. Typical scan parameters were FOV 420 × 315 mm, matrix size 256 × 192, slice thickness, 8.0 mm, flip angle 45°, TR/TE 3.1/1.4 ms and number of reconstructed phases 24 per cardiac cycle. The ascending aortic contours were manually traced in all phases. MR-AoD was calculated by dividing the difference between the maximum and minimum aortic area (in mm^2^) by the product of the minimum aortic area and the brachial pulse pressure (in mmHg).

Combined with the CMR, renal artery MR angiography was performed at baseline and throughout follow-up to evaluate the occurrence of any significant renal artery stenosis. The protocol for these measurements has been described previously [41].

CMR analyses were performed using QMass 8.1 software (Medis, Leiden, The Netherlands) for cardiac volumes, mass and function whereas MR-PWV and MR-AoD were assessed using MASS software (LUMC, Leiden, The Netherlands). All analyses were performed by the first author (V.Z.) under supervision of an experienced imaging cardiologist with more than 20 years of MR imaging experience (A.H.).

### Transthoracic echocardiography and carotid–femoral pulse wave velocity measurement

TTE was performed and analyzed according to institutional guidelines by a cardiologist specialized in echocardiography (M.G.). Data were obtained on E/e ratio, forward stroke volume index (adjusted for BSA) and valvulo-arterial impedance (Zva).

For CF-PWV, pulse wave transition time was calculated using Doppler ultrasound combined with electrocardiography tracing [42]. The distance used in the calculation was estimated as 80% of the carotid–femoral distance when measured by tape measure [43]. CF-PWV was calculated by dividing the carotid–femoral distance by the pulse wave transition time. All Doppler images were assessed by a single author (V.Z.).

### Intervention

RDN procedures were performed under conscious sedation, using fentanyl and midazolam. Unfractionated heparin was administered to achieve an activated clotting time of at least 250 s. After administration of local anesthesia, ultrasound-guided common femoral artery access was achieved and a 6 French sheath was introduced. After engaging the renal arteries, selective angiography was performed to confirm anatomical eligibility for RDN. Patients with eligible renal anatomy consequently underwent radiofrequency ablations in four quadrants of the left and right main renal arteries using the Symplicity Flex single-electrode or Symplicity Spyral multi-electrode radiofrequency RDN system (Medtronic; Minneapolis, USA).

### Baseline examinations and follow-up

**FIGURE 1:**
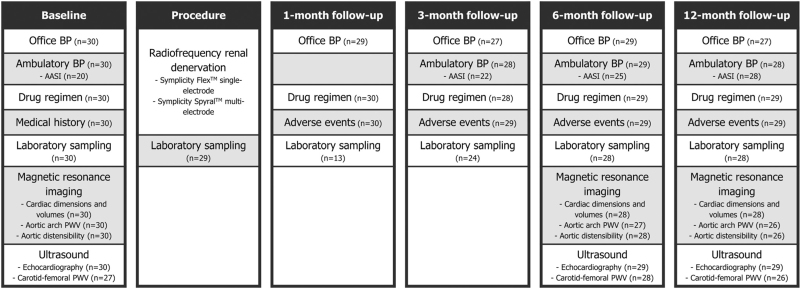
Study flowchart. BP, blood pressure; PWV, pulse wave velocity.

Patients had a preprocedural baseline visit and were followed up through clinical visits at 1, 3, 6 and 12 months after the index procedure. Evaluation of any adverse events and medication regimen, physical examination, OBP measurements and laboratory testing were performed during each visit. ABP was measured at all visits except for the 1-month visit. TTE (including CF-PWV measurement) and MR imaging (including CMR, MR-AoD/MR-PWV measurement and renal artery MR angiography) were performed at baseline and at 6-month and 12-month follow-up (Fig. 1).

### Endpoints

The primary efficacy endpoint was the temporal evolution of mean 24-h systolic ABP throughout 1-year post RDN, based on repeated measurements at baseline, 3, 6 and 12 months of follow-up. Effect modification of the primary efficacy endpoint was studied for a predefined set of baseline covariates, consisting of: vascular stiffness parameters (MR-PWV, MR-AoD, CF-PWV, AASI), CMR LV parameters (LV mass index, maximal LV wall thickness), TTE parameters (E/e ratio, forward stroke volume index, Zva), clinical parameters (age, sex, BMI, eGFR), baseline OBP [including heart rate and isolated systolic hypertension (ISH)] and ABP (mean 24-h, daytime, nighttime), antihypertensive drug defined daily doses (DDD) and procedural parameters (device type, number of ablations).

Secondary efficacy endpoints were temporal evolution of vascular stiffness parameters (MR-PWV, MR-AoD, CF-PWV, AASI), CMR LV parameters (LV mass index, maximal LV wall thickness, LV end-diastolic and end-systolic volume index, LV stroke volume index, LV ejection fraction, cardiac output), TTE parameters (E/e ratio, forward stroke volume index, Zva), BP outcomes (mean 24-h systolic and diastolic ABP, daytime systolic and diastolic ABP, nighttime systolic and diastolic ABP, systolic OBP, diastolic OBP, heart rate) and antihypertensive drug outcomes (number of drugs, DDDs, antihypertensive load index) throughout 1 year of follow-up.

The primary safety endpoint was a composite endpoint, consisting of cardiovascular death, major procedural bleeding, acute kidney injury and renal artery stenosis (whichever occurred first) up until 6 months of follow-up.

Secondary safety endpoints consisted of the individual items of the primary safety endpoint, all cardiovascular adverse events and renal function (eGFR) up until 1-year post procedure.

### Statistical analysis

Continuous variables were reported as mean ± standard deviation (SD) or median (25th to 75th percentile) for normally and non-normally distributed variables, respectively. Non-normally distributed BP values were reported in both ways to allow for comparison to previous literature. Normality was assessed using the Shapiro–Wilcoxon test and quantile–quantile plots. Categorical variables were reported as number of patients and corresponding percentages.

The primary efficacy endpoint was assessed using linear mixed-effects models with the temporal evolution of mean 24-h systolic ABP throughout 1 year of follow-up as the dependent variable and a fixed effect for time as the independent variable. Random intercepts were used to account for repeated measurements within patients, and random slopes for time were additionally included when they significantly improved the model fit (as measured with the likelihood ratio test). Results were presented as the regression coefficient for time, which can be interpreted as the annual change in the primary efficacy endpoint (mean 24-h systolic ABP) throughout 1 year post RDN [including the corresponding 95% confidence interval (CI) and *P* value].

Effect modification of the primary efficacy endpoint was studied by adding the effect modifier of interest and the interaction term [time × effect modifier] to the linear mixed-effects model. The regression coefficient for the interaction term was reported (including the corresponding 95% CI and *P* value). This coefficient can be interpreted as the additional change in mean 24-h systolic ABP over time following RDN for a one unit or level increase in the evaluated baseline effect modifier (continuous or categorical, respectively). Effect modifiers significant at alpha-level 0.20 in univariable analyses were subsequently included in multivariable linear mixed-effects models, thereby displaying corrected measures of effect. Only one interaction term was fitted per multivariable model, to preserve interpretability of the regression coefficients. To avoid collinearity, only one (surrogate) measure of arterial wall stiffness, SBP and DBP was entered in each model.

Secondary efficacy endpoints were analyzed using a similar approach as in the primary efficacy endpoint. For a subset of secondary efficacy endpoints, consisting of vascular stiffness, CMR LV and TTE endpoints, exploratory analyses were performed in case of a significant change throughout 1 year of follow-up. For these particular variables, correlation analyses between the change in mean 24-h systolic ABP and the change in the variable of interest were performed. Correlation was assessed using scatterplots and repeated measures correlation coefficients (*r*_rm_) [44].

The primary and secondary safety endpoints were assessed by presenting the number of events (percentages) for adverse event data. Renal function (eGFR) was analyzed using a similar approach as in the primary efficacy endpoint.

The current pilot study was considered exploratory and has, therefore, not been powered to detect any predetermined effect size. Unless stated otherwise, two-tailed *P* values less than 0.05 were considered statistically significant. Statistical analyses were performed using R 4.1.1 with the nlme package to perform linear mixed-effects models [45,46].

## RESULTS

### Study population

Between May 2013 and April 2019, 30 patients were enrolled. Mean age was 62.5 ± 10.7 years and 15 (50.0%) patients were female. Patients had a mean BMI of 29.4 ± 4.4 kg/m^2^ and median eGFR was 89.1 [74.3–109.5] ml/min per 1.73 m^2^. Mean 24-h ABP was 146.7/80.8 ± 13.7/12.0 and OBP was 172.4/94.6 ± 18.7/16.0 mmHg while patients were on 5.0 ± 2.4 DDDs of antihypertensive drugs. ISH was observed in 13 (43.3%) patients. Median MR-PWV was 6.8 [6.1–11.0] m/s, whereas mean CF-PWV was 8.5 ± 2.1 m/s (Table 1).

**TABLE 1:** Baseline characteristics

Variable	Patients (*n* = 30)
Clinical parameters	
Female sex [*n* (%)]	15 (50.0)
Age (years), mean ± SD	62.5 ± 10.7
BMI (kg/m^2^), mean ± SD	29.4 ± 4.4
Body surface area (m^2^), mean ± SD	2.0 ± 0.3
Estimated glomerular filtration rate (ml/min per 1.73 m^2^), median [25th to 75th percentile]	89.1 [74.3–109.5]
Smoking status	
Current smoker [*n* (%)]	6 (20.0)
Ever smoker [*n* (%)]	11 (36.7)
Medical history	
Stroke and/or transient ischemic attack [*n* (%)]	3 (10.0)
Myocardial infarction [*n* (%)]	6 (20.0)
Coronary revascularization [*n* (%)]	9 (30.0)
Diabetes mellitus type 2 [n (%)]	12 (40.0)
Ambulatory blood pressure	
Mean 24-h SBP (mmHg), mean ± SD/median [25th–75th percentile]	146.7 ± 13.7/144.5 [137.0–152.8]
Mean 24-h DBP (mmHg), mean ± SD	80.8 ± 12.0
Daytime SBP (mmHg), mean ± SD/median [25th–75th percentile]	149.8 ± 15.5/145.0 [137.3–157.5]
Daytime DBP (mmHg), mean ± SD	83.7 ± 12.8
Nighttime SBP (mmHg), mean ± SD	138.5 ± 14.9
Nighttime DBP (mmHg), mean ± SD	74.8 ± 13.6
Ambulatory arterial stiffness index, mean ± SD	0.53 ± 0.13
Office blood pressure	
SBP (mmHg), mean ± SD	172.4 ± 18.7
DBP (mmHg), mean ± SD	94.6 ± 16.0
Heart rate (beats per minute), median [25th–75th percentile]	67.5 [60.0–75.5]
Isolated systolic hypertension [*n* (%)]	13 (43.3)
Antihypertensive drug treatment – summary measures	
Defined daily doses, mean ± SD	5.0 ± 2.4
Antihypertensive load index, median [25th–75th percentile]	2.2 [1.8–3.2]
Total number of drugs, mean ± SD	3.4 ± 1.3
Intolerance to ≥ 3 classes of antihypertensive drugs [*n* (%)]	4 (13.3)
Antihypertensive drug treatment – individual classes	
Thiazide diuretic [*n* (%)]	23 (76.7)
Angiotensin-converting enzyme inhibitor [*n* (%)]	5 (16.7)
Angiotensin receptor blocker [*n* (%)]	23 (76.7)
Calcium channel blocker [*n* (%)]	23 (76.7)
Aldosterone antagonist [*n* (%)]	6 (20.0)
Alpha antagonist [*n* (%)]	10 (33.3)
Vasodilator [*n* (%)]	4 (13.3)
Direct renin inhibitor [*n* (%)]	1 (3.3)
Cardiovascular magnetic resonance imaging	
LV mass index (g/m^2^), mean ± SD	66.8 ± 15.4
Maximal wall thickness (mm), mean ± SD	12.3 ± 2.7
LV end-diastolic volume index (ml/m^2^), median [25th–75th percentile]	74.3 [69.9–86.9]
LV end-systolic volume index (ml/m^2^), median [25th–75th percentile]	28.6 [24.0–33.1]
LV ejection fraction (%), mean ± SD	62.5 ± 7.7
LV stroke volume index (ml/m^2^), mean ± SD	48.4 ± 6.8
Cardiac output (l/min), median [25th–75th percentile]	6.3 [5.6–7.3]
Echocardiography	
*E*/*é* ratio, mean ± SD	14.6 ± 5.2
Forward stroke volume index (ml/m^2^), mean ± SD	41.2 ± 10.2
Valvulo-arterial impedance (mmHg/ml/m^2^), mean ± SD	4.6 ± 1.2
Vascular parameters	
MR-pulse wave velocity (m/s), median [25th–75th percentile]	6.8 [6.1–11.0]
MR-aortic distensibility (10^–3^/mmHg), median [25th–75th percentile]	1.4 [0.9–1.8]
CF-pulse wave velocity (m/s), mean ± SD	8.5 ± 2.1
Procedural characteristics	
Procedure time (minutes), median [25th–75th percentile]	58.5 [48.5–70.0]
Contrast volume used (ml), median [25th–75th percentile]	70.0 [50.0–120.0]
Radiofrequency renal denervation device	
Symplicity Flex [*n* (%)]	11 (36.7)
Symplicity Spyral [*n* (%)]	19 (63.3)
Total number of emissions bilaterally, median [25th–75th percentile]	17.5 [10.0–23.8]
Right renal emissions, median [25th–75th percentile]	6.0 [5.0–11.0]
Left renal emissions, median [25th–75th percentile]	9 [5.0–12.8]

CF, carotid–femoral; LV, left ventricular; MR, magnetic resonance; SD, standard deviation.

### Procedural characteristics

Median procedural time was 58.5 [48.5–70.0] minutes in which 17.5 [10.0–23.8] emissions were performed bilaterally. Eleven patients (36.7%) were treated with the Symplicity Flex radiofrequency RDN device whereas 19 patients (63.3%) were treated with the Symplicity Spyral radiofrequency RDN device (Table 1).

### Primary efficacy endpoint

**FIGURE 2:**
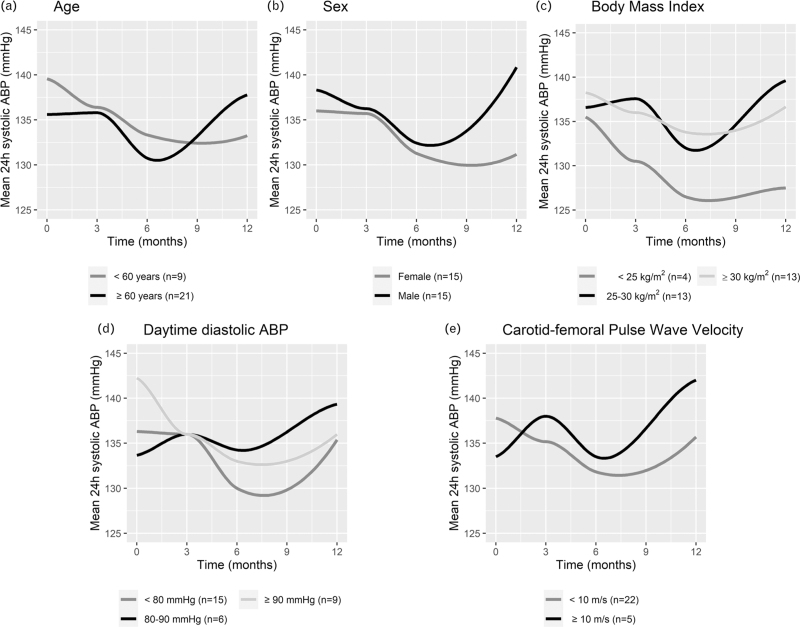
Mean 24-h ambulatory blood pressure over time post renal denervation for different levels of baseline effect modifiers. ABP, ambulatory blood pressure.

Mean 24-h systolic ABP decreased with −8.4 mmHg/year (95% CI −14.5 to −2.3; *P* = 0.007) post RDN (Table 3). Baseline CF-PWV was identified as an independent effect modifier of the change in mean 24-h systolic ABP post RDN (+2.7 mmHg/year in mean 24-h systolic ABP per m/s increase in baseline CF-PWV; 95% CI 0.3–5.1; *P* = 0.03) after correction for age, sex, BMI and heart rate. Similarly, variables that emerged as independent effect modifiers were daytime diastolic ABP (−0.4 mmHg/year per mmHg; 95% CI −0.8 to 0.0; *P* = 0.03), age (+0.6 mmHg/year per year of age; 95% CI 0.2–1.0; *P* = 0.006), female sex (−14.0 mmHg/year as compared with male; 95% CI −23.1 to −5.0; *P* = 0.003) and BMI (+1.2 mmHg/year per kg/m^2^; 95% CI 0.1–2.2; *P* = 0.04). MR-PWV did not emerge as a significant effect modifier (+1.1 mmHg/year per m/s increase in baseline MR-PWV; 95% CI −0.1 to 2.3; *P* = 0.07) (Table 2 and Fig. 2). Similar findings were observed in univariable analyses. None of the other evaluated baseline covariates, including RDN device type, demonstrated any effect modification (Supplemental Table 1).

**TABLE 2:** Multivariable analysis of baseline effect modifiers of change over time in mean 24-h systolic ambulatory blood pressure post renal denervation

Baseline covariates	Change in mean 24-h systolic ABP post renal denervation in mmHg/year (95% CI)	*P* value
Clinical parameters		
Age (years)	0.6 (0.2–1.0)	0.006
Female sex (as compared with male)	−14.0 (−23.1 to −5.0)	0.003
BMI (kg/m^2^)	1.2 (0.1–2.2)	0.04
Office blood pressure		
Heart rate (beats per minute)	−0.2 (−0.4 to 0.0)	0.08
Ambulatory blood pressure		
Mean 24-h DBP (mmHg)	−0.3 (−0.7 to 0.0)	0.08
Daytime SBP (mmHg)	−0.2 (−0.5 to 0.1)	0.17
Daytime DBP (mmHg)	−0.4 (−0.8 to 0.0)	0.03
Vascular parameters		
MR-pulse wave velocity (m/s)	1.1 (−0.1 to 2.3)	0.07
CF-pulse wave velocity (m/s)	2.7 (0.3–5.1)	0.03

All models contained fixed effects for time, age, sex, BMI, heart rate and the variable of interest, as well as an interaction term [time × variable of interest]. Random effects were used to account for repeated measurements of the variable of interest within patients. The regression coefficient for this interaction term was presented (including CIs and *P* values). For continuous effect modifiers, the additional change in mmHg/year post renal denervation was presented per increase of one unit in the effect modifier. For categorical effect modifiers, the additional change in mmHg/year post renal denervation was presented as compared to a given reference level of the effect modifier. ABP, ambulatory blood pressure; CI, confidence interval; MR, magnetic resonance imaging; US, ultrasound.

### Secondary efficacy endpoints

**FIGURE 3:**
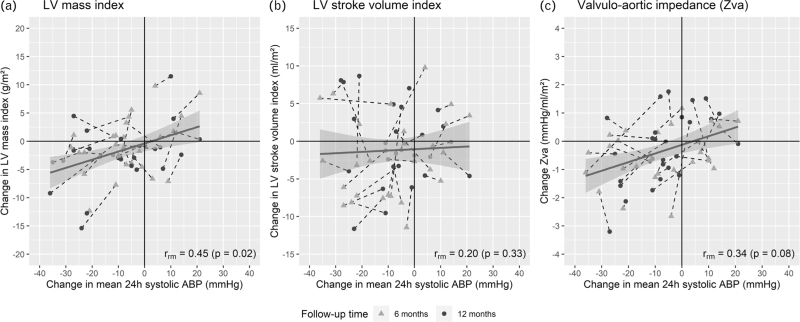
Correlation between change in mean 24-h ambulatory blood pressure and (a) change in left ventricular mass index, (b) left ventricular stroke volume index and (c) valvulo-aortic impedance (Zva) at 6 and 12 months post renal denervation. ABP, ambulatory blood pressure; LV, left ventricular; *r*_rm_, repeated measures correlation.

Throughout follow-up, significant reductions were observed in LV mass index (−2.3 g/m^2^ per year; 95% CI −4.0 to −0.5; *P* = 0.01), LV stroke volume index (−2.1 ml/m^2^ per year; 95% CI −4.1 to −0,1; *P* = 0.04) and Zva (−0.5 mmHg/ml per m^2^ per year; 95% CI −1.0 to −0.1; *P* = 0.01). Furthermore, reductions were observed in mean 24-h diastolic ABP (−5.7 mmHg/year; 95% CI −8.4 to −3.0; *P* < 0.001), systolic OBP (−9.0 mmHg/year; 95% CI −16.2 to −1.7; *P* = 0.02) and diastolic OBP (−8.0 mmHg/year; 95% CI −11.7 to −4.4; *P* < 0.001), daytime systolic ABP (−8.4 mmHg/year; 95% CI −15.7 to −1.1; *P* = 0.02) and diastolic ABP (−5.9 mmHg/year; 95% CI −9.7 to −2.2; *P* = 0.003), nighttime systolic ABP (−7.5 mmHg/year; 95% CI −12.0 to −2.9; *P* = 0.002) and diastolic ABP (−4.6 mmHg/year; 95% CI −7.9 to −1.4; *P* = 0.005) throughout 1 year of follow-up. No changes over time were observed in other secondary outcomes (Table 3). The change in mean 24-h systolic ABP was correlated with the change in LV mass index between baseline and 6 and 12-month follow-up (*r*_rm_ = 0.45; *P* = 0.02) (Fig. 3).

**TABLE 3:** Change in outcome measures during 1 year after renal denervation

Variable	Modelled change post renal denervation per year (95% CI)	*P* value
Ambulatory blood pressure		
Mean 24-h systolic blood pressure (mmHg)	−8.4 (−14.5 to −2.3)	0.007
Mean 24-h diastolic blood pressure (mmHg)	−5.7 (−8.4 to −3.0)	<0.001
Daytime systolic blood pressure (mmHg)	−8.4 (−15.7 to −1.1)	0.02
Daytime diastolic blood pressure (mmHg)	−5.9 (−9.7 to −2.2)	0.003
Nighttime systolic blood pressure (mmHg)	−7.5 (−12.0 to −2.9)	0.002
Nighttime diastolic blood pressure (mmHg)	−4.6 (−7.9 to −1.4)	0.005
Ambulatory arterial stiffness index (subset of patients; *n* = 20)	0.0 (−0.1 to 0.0)	0.17
Office blood pressure		
Systolic blood pressure (mmHg)	−9.0 (−16.2 to −1.7)	0.02
Diastolic blood pressure (mmHg)	−8.0 (−11.7 to −4.4)	<0.001
Heart rate (beats per minute)	−1.8 (−5.1 to 1.5)	0.28
Antihypertensive drug treatment		
Defined daily doses	0.0 (−0.4 to 0.3)	0.79
Antihypertensive load index	0.0 (−0.2 to 0.2)	0.94
Total number of drugs	0.0 (−0.2 to 0.3)	0.75
Vascular parameters		
MR-pulse wave velocity (m/s)	0.1 (−1.9 to 2.1)	0.92
MR-aortic distensibility (10^–3^/mmHg)	0.0 (−0.4 to 0.4)	0.84
CF-pulse wave velocity (m/s)	0.5 (−0.6 to 1.5)	0.37
Cardiovascular magnetic resonance		
LV mass index (g/m^2^)	−2.3 (−4.0 to −0.5)	0.01
Maximal wall thickness (mm)	0.1 (−0.5 to 0.7)	0.74
LV end-diastolic volume index (ml/m^2^)	−2.5 (−5.9 to 1.0)	0.16
LV end-systolic volume index (ml/m^2^)	−0.4 (−2.8 to 2.0)	0.75
LV stroke volume index (ml/m^2^)	−2.1 (−4.1 to −0.1)	0.04
LV ejection fraction (%)	−1.2 (−2.9 to 0.5)	0.17
Cardiac output (l/min)	−0.5 (−1.2 to 0.1)	0.10
Echocardiography		
*E*/*é* ratio	−1.0 (−2.5 to 0.5)	0.19
Forward stroke volume index (ml/m^2^)	1.0 (−1.6 to 3.6)	0.45
Valvulo-arterial impedance (mmHg/ml/m^2^)	−0.5 (−1.0 to −0.1)	0.01

LV, left ventricular; MR, magnetic resonance; SD, standard deviation; US, ultrasound.

### Primary and secondary safety endpoints

The primary safety endpoint of this study occurred in two (6.7%) patients. One patient (3.3%) died one-and-a-half months after the procedure, most likely because of a cardiac arrhythmia. Another patient (3.3%) had a retroperitoneal bleeding, which required no additional intervention. This patient received intravenous fluid therapy as well as one unit of packed red blood cells and was discharged from the hospital after 4 days in good clinical condition. No newly acquired renal artery stenosis and renal failure occurred within 1 year after the index procedure. Within 1 year of follow-up, four patients (13.3%) presented with a hypertensive emergency and two patients (6.7%) had a stroke or transient ischemic attack. Coronary revascularization was performed in two patients (6.7%), of which one (3.3%) presented with a myocardial infarction (Table 4). Renal function (eGFR) remained stable throughout 1-year post RDN (−4.3 ml/min per 1.73 m^2^ per year; 95% CI –10.6 to 2.0; *P* = 0.18).

**TABLE 4:** Safety endpoints

Clinical endpoint	Patients (*n* = 30)
Primary safety endpoint (cardiovascular death, major procedural bleeding, acute kidney injury or renal artery stenosis at 6 months) [*n* (%)]	2 (6.7)
Cardiovascular death at 6 months [*n* (%)]	1 (3.3)
Major procedural bleeding [*n* (%)]	1 (3.3)
Newly acquired renal artery stenosis and/or repeat renal artery intervention at 6 months [*n* (%)]	0 (0.0)
Acute kidney injury at 6 months [*n* (%)]	0 (0.0)
Secondary safety endpoints (12 months)	
Cardiovascular death [*n* (%)]	1 (3.3)
Newly acquired renal artery stenosis and/or repeat renal artery intervention [*n* (%)]	0 (0.0)
Development of renal failure or requirement of dialysis [*n* (%)]	0 (0.0)
Hospitalization for hypertensive emergency [*n* (%)]	4 (13.3)
Stroke or transient ischemic attack [*n* (%)]	2 (6.7)
Myocardial infarction [*n* (%)]	1 (3.3)
Coronary revascularization [*n* (%)]	2 (6.7)

## DISCUSSION

This single-center pilot study aimed to evaluate the potential of noninvasively measured arterial stiffness indices to predict the response to RDN in a cohort of patients with resistant hypertension. We demonstrated that baseline CF-PWV was an independent effect modifier of BP response following RDN. Moreover, we confirmed the effect-modifying ability of previously identified baseline characteristics such as age, sex and BMI. Finally, we demonstrated a significant reduction in LV mass index post RDN.

In contrast to MR-PWV and MR-AoD, CF-PWV significantly correlated with the magnitude of the RDN-induced BP response, which could be explained by a difference in path length used in each measurement. For CF-PWV, the path measured includes a substantial part of the large arteries, whereas for MR-PWV and MR-AoD, only the aortic arch and ascending aorta, respectively, are included in the calculation. As a result, the measurement error for MR-PWV will be conceptually larger as compared with CF-PWV because of the smaller magnitude of the distance and time values. This conceptual difference could result in a lower level of agreement between MR-PWV and invasive PWV as compared with CF-PWV and invasive PWV, which could be considered an inherent limitation of this diagnostic modality. This finding was supported by previous work demonstrating a stronger correlation with invasive PWV for CF-PWV as compared with MR-PWV [47,48]. Similarly, we found that ISH, which is also considered a surrogate marker of arterial stiffness, was not an effect modifier of the response to RDN. Based on the present findings, CF-PWV, being a validated alternative to invasive PWV assessment, emerged as the most relevant metric for arterial stiffness and showed to independently predict response to RDN. Of note, MR-PWV measurements including the aortic arch up until the diaphragm or femoral bifurcation were not routinely performed in this study.

The current study confirmed previous literature demonstrating no association between MR-PWV and the BP response following RDN [32]. However, our findings on CF-PWV and MR-AoD were in contrast with previous work, which stated that MR-AoD was associated with the magnitude of the BP response to RDN, whereas CF-PWV was not [31,32]. These findings likely reflect the limited sample size of all studies on the topic, together with differences in study design and population. Specifically, the patients in the current study had lower levels of baseline CF-PWV (8.5 vs. 11.2 m/s) and MR-PWV (6.8 vs. 8.5 m/s) and were prescribed a lower number of antihypertensive drugs (3.4 vs. 5.0 drugs) as compared with the previous study, while age and mean 24-h systolic ABP were similar [32]. These discrepancies could be explained by a difference in severity of hypertension and subsequent hypertension-mediated organ damage. Of note, the baseline level of arterial stiffness in our study was comparable to levels of arterial stiffness in a healthy population of the same age [49,50]. Taken together, these findings suggest that variation in the severity of hypertension and arterial stiffness in the patient population of interest could affect the additive value of incorporating noninvasive arterial stiffness assessment in the preprocedural work-up for RDN. Of note, no invasive PWV measurements were performed in the current study, precluding any comparison to previous literature on that topic.

The more pronounced focus on longitudinal data in our study could have resulted in conclusions different from those reported in previous studies. Whereas previous work estimated an outcome variable at a single fixed time point (i.e. 3 months), we included four repeated measures of the outcome variable mean 24-h systolic ABP to allow for more adequate distinction between sustained BP changes over time and natural BP variation [32]. This technique allowed for a larger number of analyzable outcome measurements. Furthermore, we refrained from dichotomizing our outcome variable (i.e. the concept of ‘responders’ or ‘super-responders’) but rather implemented continuous variables to maintain statistical power in this unpowered pilot study and to prevent inflated coefficients with limited clinical implications.

With respect to the effect of other effect modifiers, our observations largely confirmed the findings of previous work and should be interpreted in light of the small sample size of the present study. For age, the 12-month change in mean 24-h systolic ABP to RDN in our dataset was reduced by 0.6 mmHg per year of age increase at baseline, similar to an observed effect of only 0.1 mmHg in the literature [16]. Furthermore, female sex was associated with a −14.0 mmHg additional reduction in systolic ABP post RDN as compared with male sex, while only an effect of −0.9 mmHg was observed previously [16]. Finally, higher baseline BMI demonstrated to diminish the effect of RDN with 1.2 mmHg per kg/m^2^, relating to previously observed effect sizes of −0.3 to 0.7 mmHg [13,16].

During 1 year of follow-up post RDN, we observed a decrease in CMR LV mass index of −2.3 g/m^2^, which is line with previously observed effect sizes (−2.6 g/m^2^) [37]. These findings may reflect the effect of reduced sympathetic tone on (partial) reversibility of LV hypertrophy, with either a direct effect and/or an indirect effect through BP reduction. Although sympathetic nerve activity was not measured in this study, we did observe a significant correlation between the reduction in LV mass index and mean 24-h systolic ABP. With respect to PWV and AoD, no significant reductions post RDN were observed in the current study, which conflicts with previous data [34–36]. This finding could be attributed to the more favorable patient profile in the present study, including younger patients with lower degrees of arterial stiffness.

With respect to BP reduction, RDN demonstrated to significantly reduce mean 24-h systolic ABP (−8.4 mmHg) and diastolic ABP (−5.7 mmHg) as well as systolic OBP (−9.0 mmHg) and diastolic OBP (−8.0 mmHg) throughout 1 year of follow-up. These findings are consistent with the changes in mean 24-h systolic ABP (−8.5 to −9.0 mmHg) and diastolic ABP (−5.4 to −6.0 mmHg) as well as systolic OBP (−9.0 to −9.4 mmHg) and diastolic OBP (−5.0 to −5.2 mmHg) following RDN in the treatment arm of randomized sham-controlled trials [6,8].

### Limitations

First, this pilot study was not statistically powered to detect a certain predetermined effect. As a result, we cannot rule out that our inferences have been influenced by a lack of statistical power. Furthermore, effect modification was tested for a wide variety of variables in this explorative study, consequently increasing the risk of false-positive findings. Second, this study lacked a comparator arm as well as direct measurements of sympathetic nerve tone, precluding statements on any causal relationships. For BP specifically, we cannot rule out a placebo effect as previous RDN trials reported significant BP reductions also in the control group [6,8,9]. Consequently, larger, adequately powered, randomized studies will be needed to draw more robust conclusions on the role of noninvasively measured arterial stiffness in identifying potential responders to RDN before implementation of such indices into routine clinical practice. Third, we were able to rule out a Hawthorne effect related to drug prescriptions, as antihypertensive drug burden remained stable over time. However, as this study lacked drug adherence testing, we cannot rule out that any change in adherence has influenced our results. Finally, the inclusion rate in this study was hampered by the negative results of the SYMPLICITY HTN-3 trial and the manufacturing of the Symplicity Flex catheter was discontinued during our enrollment period, mandating us to change to the use of the successive Symplicity Spyral RDN system (Medtronic; Minneapolis, Minnesota, USA).

In conclusion, RDN demonstrated to lower mean 24-h systolic ABP throughout 1 year of follow-up. Higher baseline CF-PWV was independently associated with a smaller reduction in mean 24-h systolic ABP response post RDN. In addition, higher baseline age and BMI were also independently associated with a smaller reduction in systolic ABP following RDN. In contrast, female sex was associated with a larger reduction in systolic ABP. MR-derived arterial stiffness indices did not predict the response to RDN.

## ACKNOWLEDGEMENTS

Sources of support for this work: this study was funded by Medtronic.

### Conflicts of interest

V.Z. received institutional grant support from ReCor Medical and Medtronic. N.v.M. received institutional grant support from Abbott Vascular, Boston Scientific, Biotronik, Edwards Lifesciences, Medtronic, PulseCath BV, Abiomed, Daiichi Sankyo, Materialise, Pie Medical and Siemens. J.D. received institutional grant support from Astra Zeneca, Abbott Vascular, Boston Scientific, ACIST Medical, Medtronic, Microport, Pie Medical, and ReCor medical. All other authors declare no competing interests.

## Supplementary Material

Supplemental Digital Content
