# Supplementary material for: Association between renal sympathetic denervation and arterial stiffness: the ASORAS study
Source: J Hypertens. 2023 Jan 19;41(3):476–85. doi: 10.1097/HJH.0000000000003361 (PMC9894147; doi:10.1097/HJH.0000000000003361)
Supplement: Supplemental Digital Content [file jhype-41-476-s001.pdf]

Supplemental Material

Supplemental Table 1. Univariable effect modifiers of change in mean 24h ambulatory systolic blood pressure post renal denervation

Baseline covariates	Change in mean 24h systolic ABP post renal denervation in mmHg/year (95% CI)	P-value
**Clinical parameters**		
Age (years)	0.6 (0.1, 1.1)	0.02
Female sex (as compared to male)	-14.0 (-23.1, -5.0)	0.003
Body Mass Index (kg/m^2)	1.2 (-0.2, 2.6)	0.09
Estimated Glomerular Filtration Rate (ml/min/1.73 m^2)	-0.1 (-0.3, 0.1)	0.52
**Ambulatory blood pressure**		
Mean 24h systolic blood pressure (mmHg)	-0.1 (-0.5, 0.2)	0.49
Mean 24h diastolic blood pressure (mmHg)	-0.4 (-0.8, 0.1)	0.16
Daytime systolic blood pressure (mmHg)	-0.2 (-0.6, 0.1)	0.14
Daytime diastolic blood pressure (mmHg)	-0.4 (-0.9, 0.1)	0.09
Nighttime systolic blood pressure (mmHg)	0.2 (-0.2, 0.6)	0.41
Nighttime diastolic blood pressure (mmHg)	-0.2 (-0.6, 0.3)	0.50
Ambulatory Arterial Stiffness Index	3.2 (-40.9, 47.3)	0.88
**Office blood pressure**		
Systolic blood pressure (mmHg)	0.1 (-0.3, 0.4)	0.62
Diastolic blood pressure (mmHg)	0.1 (-0.3, 0.5)	0.62
Heart rate (beats per minute)	-0.2 (-0.5, 0.1)	0.16
Isolated systolic hypertension	-0.5 (-13.0, 12.0)	0.93

**Antihypertensive drug treatment**		
Defined Daily Doses	-0.3 (-3.0, 2.4)	0.82
**Cardiovascular magnetic resonance**		
LV mass index (g/m^2)	0.1 (-0.2, 0.4)	0.40
Maximal wall thickness (mm)	0.7 (-1.6, 3.0)	0.56
**Echocardiography**		
E/e′ ratio	0.1 (-1.1, 1.4)	0.83
Forward stroke volume index (ml/m^2)	0.0 (-0.7, 0.6)	0.87
Valvulo-arterial impedance ($mmHg/ml/m^2$)	-0.2 (-5.2, 4.8)	0.94
**Vascular parameters**		
MR-Pulse Wave Velocity (m/s)	1.1 (-0.1, 2.3)	0.07
MR-Aortic Distensibility (10^{-3} $mmHg^{-1}$)	-2.0 (-6.8, 2.8)	0.40
CF-Pulse Wave Velocity (m/s)	2.8 (-0.3, 5.8)	0.07
**Procedural characteristics**		
Spyral™ device (as compared to Flex™ device)	4.9 (-7.7, 17.5)	0.44
Total number of ablations	0.3 (-0.4, 1.1)	0.39

*All models contained fixed effects for time, age, sex, body mass index, heart rate and the variable of interest, as well as an interaction term [time * variable of interest]. Random effects were used to account for repeated measurements of the variable of interest within patients.*

The regression coefficient for this interaction term was presented (including CIs and P-values). For continuous effect modifiers, the additional change in mmHg/year post renal denervation was presented per increase of one unit in the effect modifier. For categorical effect modifiers, the additional change in mmHg/year post renal denervation was presented as compared to a given reference level of the effect modifier.

LV, Left Ventricular. MR, Magnetic Resonance. ABP, Ambulatory Blood Pressure. SD, Standard Deviation. US, Ultrasound.
